# Obesity Takes Its Toll on Visceral Pain: High-Fat Diet Induces Toll-Like Receptor 4-Dependent Visceral Hypersensitivity

**DOI:** 10.1371/journal.pone.0155367

**Published:** 2016-05-09

**Authors:** Mónica Tramullas, Beate C. Finger, Timothy G. Dinan, John F. Cryan

**Affiliations:** 1 APC Microbiome Institute, University College Cork, Cork, Ireland; 2 School of Pharmacy, University College Cork, Cork, Ireland; 3 Department of Psychiatry & Neurobehavioural Science, University College Cork, Cork, Ireland; 4 Department of Anatomy & Neuroscience, University College Cork, Cork, Ireland; Monash University, AUSTRALIA

## Abstract

Exposure to high-fat diet induces both, peripheral and central alterations in TLR4 expression. Moreover, functional TLR4 is required for the development of high-fat diet-induced obesity. Recently, central alterations in TLR4 expression have been associated with the modulation of visceral pain. However, it remains unknown whether there is a functional interaction between the role of TLR4 in diet-induced obesity and in visceral pain. In the present study we investigated the impact of long-term exposure to high-fat diet on visceral pain perception and on the levels of TLR4 and Cd11b (a microglial cell marker) protein expression in the prefrontal cortex (PFC) and hippocampus. Peripheral alterations in TLR4 were assessed following the stimulation of spleenocytes with the TLR4-agonist LPS. Finally, we evaluated the effect of blocking TLR4 on visceral nociception, by administering TAK-242, a selective TLR4-antagonist. Our results demonstrated that exposure to high-fat diet induced visceral hypersensitivity. In parallel, enhanced TLR4 expression and microglia activation were found in brain areas related to visceral pain, the PFC and the hippocampus. Likewise, peripheral TLR4 activity was increased following long-term exposure to high-fat diet, resulting in an increased level of pro-inflammatory cytokines. Finally, TLR4 blockage counteracted the hyperalgesic phenotype present in mice fed on high-fat diet. Our data reveal a role for TLR4 in visceral pain modulation in a model of diet-induced obesity, and point to TLR4 as a potential therapeutic target for the development of drugs to treat visceral hypersensitivity present in pathologies associated to fat diet consumption.

## Introduction

Obesity has been associated with inflammatory processes in the periphery as well as in the central nervous system [1–5]. Several reports show alterations in central and peripheral immune signaling in a widely-used mouse model of obesity, the diet-induced obese mouse [6–9]. Moreover, studies have implicated a role for Toll-like receptor 4 in these high-fat diet-induced inflammatory processes. An increased release of systemic endotoxins in response to the consumption of fat-rich diets [10, 11] and the saturated fatty acids themselves [12–14] are mediating the inflammatory response via TLR4 activation. Toll like receptors (TLRs) are a family of pattern-recognition receptors in the innate immune system. TLR4 is selectively activated by lipopolysaccharide (LPS), a gram-negative bacterial cell wall component, which upon binding activates several immune signaling cascades. Within the CNS TLR4 is also present and is predominately expressed in microglial cells [15], which represent the first line of defense, acting as a sensor of pathological events [16]. Long-term exposure to diets high in fat content increases peripheral TLR4 expression and cause increased circulation of LPS [6, 10]. The link between diet-induced obesity and TLR4 was also described in studies demonstrating that TLR4 knockout mice are resistant to develop a diet-induced phenotype when fed high-fat diet for several month [17, 18].

We have recently shown that both, absence of functional TLR4 and pharmacological blockade of TLR4 via peripheral, intracerebroventricular or intra-PFC administration of a small-molecule TLR4 antagonist, reduces visceral pain and prevents the development of stress-induced visceral hypersensitivity. In addition, increased expression of TLR4 is coupled with enhanced microglia activation in the PFC in our model of stress-induced visceral pain [19]. In the same vein we have shown that LPS increases visceral hypersensitivity [20].

In the present study, we investigated whether there is a functional link between the role of TLR4 in diet-induced obesity and the reported modulatory function of TLR4 in visceral hypersensitivity. To this end using a model of diet-induced obesity, where mice are exposed to high-fat diet for several months, we assessed visceral sensitivity and alterations in expression of TLR4 and microglia within the CNS, in the PFC and the hippocampus, two areas implicated in pain processing [21]. Alterations in functional TLR4 activity in the periphery were also investigated. Finally, we sought to confirm whether TLR4 regulates high-fat diet-induced visceral pain by administration of a small-molecule and selective TLR4 antagonist, TAK-242.

## Materials and Methods

### 2.1 Animals

Diet-induced obese model male C57BL/6J mice (n = 80, age 3 weeks at arrival) were used as previously described [22, 23]. All groups were split into cohorts that were used for behavioral testing (colorectal distension) and sample groups (naïve) for protein level and spleen cytokines analysis, housed in groups of five. Water and food were available *ad libitum* to all mice throughout the whole study. The holding room was temperature (21±1°C) and humidity (55±10%) controlled and under a 12-hour light/dark cycle (lights on 7.00am). All animals were supplied by Harlan (UK). All experiments were conducted in accordance with the European Directive 2012/707/EU and approved by the Animal Experimentation Ethics Committee of University College Cork. All efforts were made to minimize animal suffering and to reduce the number of animals used.

### 2.2 Diet-induced obesity (DIO)

After arrival, the 3-week old C57BL/6J mice received high-fat (n = 40; 45% kcal from fat (lard 87.7%, soybean oil 12.3%); Research Diets; #D12451, New Brunswick, New Jersey, USA) or low-fat diet (n = 40; 10%kcal from fat (soybean oil 56%, lard 44%), Research Diets, #D12450B, New Brunswick, New Jersey, USA;) respectively throughout the whole study. As described previously, feeding was carried out for 16 weeks to induce adiposity in the obesity prone C57BL/6J strain [22].

### 2.3 Colorectal distension (CRD)

Colorectal distension (CRD) is a paradigm frequently used in animals [24] and humans [25] to assess visceral pain.

CRD was carried out as described previously [26, 27]. Briefly, mice were lightly anesthetized with isoflurane (2% vapor in oxygen; IsoFlo^®^, Abbott, UK) and a balloon with a connecting catheter was inserted into the colon, 0.5 cm proximal to the anus. The unrestrained mice were allowed to recover for 10 minutes before starting the CRD procedure. At the end of the experiments, the balloon was carefully removed and the animals were returned to their home cages. CRD produces contractions of the abdominal musculature, termed the visceromotor response (VMR). From previous reports in rats and mice, pressure oscillations during the inflation of the intracolonic balloon are closely related to the presence of abdominal muscle contractions associated to CRD and therefore, intraballoon pressure changes are a valid method to assess the VMR associated to the CRD [28, 29]. An ascending phasic distension (from 10 to 80 mmHg) paradigm was used and thus, VMR to distension was quantified as pressure changes observed within the colonic distending balloon during the colorectal distension procedure. Additionally, for every animal, pain threshold was defined as the pressure of the distending pulse at which the first painful response was evoked and defined as the pressure which exceeded the mean baseline activity plus three times the standard deviation.

### 2.4 Drug-administration

TAK-242 (Discovery Fine Chemicals, UK), a small-molecule and selective TLR4 antagonist [30], was dissolved in a fat emulsion (50% soybean oil in saline) and administered intravenously via the tail vein (i.v., 10 mg/kg) one hour prior to CRD. We have shown previously [19], that not only central but also peripheral administration of this TLR4 antagonist reduces visceral pain levels in animal models of visceral hypersensitivity.

### 2.5 Body composition analysis

Body composition analysis was carried out as described previously [22]. Measurements of adipose and lean mass, expressed as percentage, were performed in a three-minute trial using the Minispec^®^ NMR analyzer (mq 7.5; Bruker Optics, USA).

### 2.6 Western blot

At the end of 16 weeks, CRD-naïve mice were sacrificed without anesthesia and the dissected brains and spinal cords snap-frozen and stored at -80°C. Western blot was performed as previously described [31]. Whole-cell lysates were prepared from the prefrontal cortex, the hippocampus and the lumbar region of the spinal cord and total protein was determined using the Quan-iT^™^ protein assay kit (Invitrogen, Ireland). Equal amounts of protein were subjected to electrophoresis on 4–12% gradient gels (NuPAGE, Invitrogen, Ireland) and transferred to a polyvinylidene difluoride membrane (Bio Rad, Ireland). Membranes were then incubated with rabbit anti-TLR4 (Abcam, Ireland) and rabbit anti-CD11b (Novus Biologicals, UK). Spleen lysates were used as a positive control for TLR4-antibody detection. Inmunoreactivity was detected with Pierce ECL detection reagent (Thermo Scientific, IL, USA) and visualized using a luminescent image analyzer (LAS-3000, Fugifilm, Ireland). Optical density of the immunoreactive bands was quantified using ImageJ software and normalized to mouse anti-β actin (Sigma, Ireland).

### 2.7 Spleen cytokine assays

Mice were sacrificed without anesthesia and the spleens were dissected under sterile conditions in culture medium (RPMI 1640; Sigma–Aldrich, Ireland) with 10%FCS. Spleen cells were counted in a hemocytometer (Marienfeld) and a spleen cell suspension at 2x10^6^ was prepared. Spleen cells (100μL per well, 2x10^6^ cells/well) were added to 96-well plates and cultured in a 37°C incubator with 5% CO2. Each sample was cultured in triplicate with 1μg/ml of lipopolysaccharide (LPS, Sigma–Aldrich, Ireland) or saline (untreated) for 24h. 96-well plates were centrifuged at 1300 r.p.m. for 5 min and cell culture supernatant was collected and stored at −80°C until assayed. Spleen culture supernatant samples were analysed for IL6 and TNF-α using custom mouse Multi-spot^®^ 96-well plates (MSD, Gaithersburg, MD, USA) as previously described [19]. ELISA plates were analysed using the Sector 2400 imager from Mesoscale Discovery. This is an ultra-sensitive method which has a detection limit for IL-6 of 0.3 pg/ml and TNFαof 0.3 pg/ml.

### 2.8 Statistical analysis

Statistical differences between groups were analyzed by repeated measures one- or two-way analysis of variance (ANOVA) followed by Bonferroni’s post-hoc test. Independent-sample t-tests were used to compare two independent groups. All tests were performed at a significance level of p<0.05. All analysis was carried out using SPSS 18.0 for windows (SPSS Inc., Chicago, USA). All graphs show mean values ± standard error of the mean (SEM) with * p<0.05; ** p<0.01; *** p<0.001.

## Results

### 3.1 Diet-induced obesity

**Fig 1:**
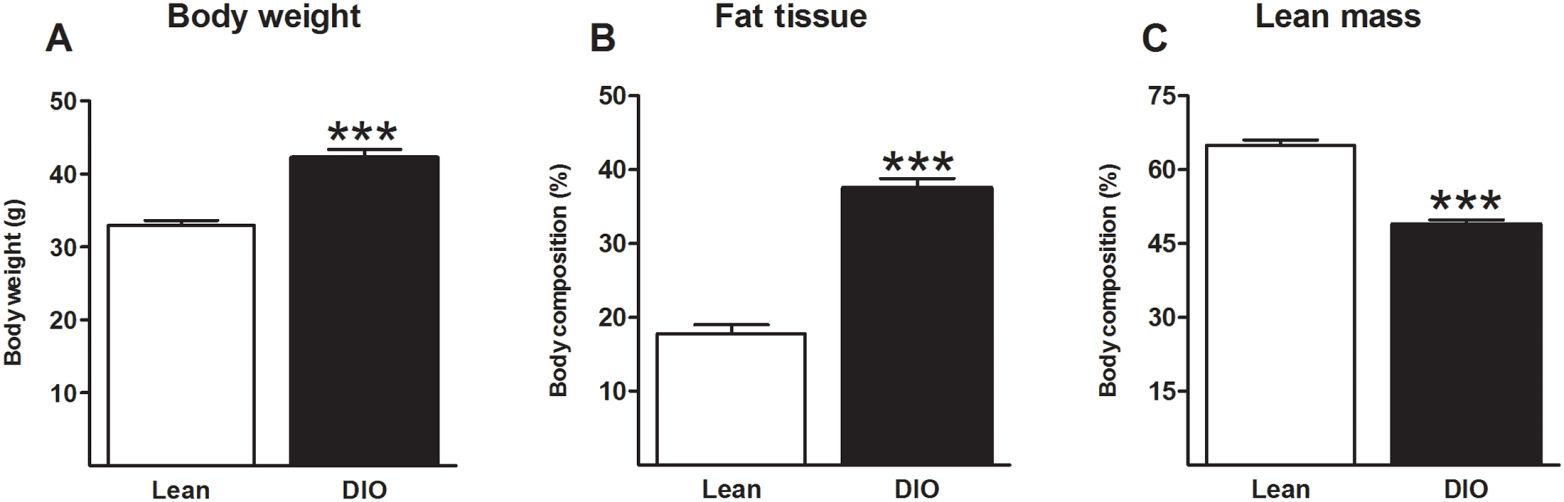
Diet-induced obesity. Long-term exposure to high-fat diet increases body-weight (A), percentage of adipose body mass (B) and a relative decrease in percentage of lean body mass (C) in diet-induced obese (DIO) mice compared to lean controls fed on a low-fat diet. *** p<0.001; independent sample t-test; data represent mean ± standard error of the mean (SEM).

Mice fed on the high-fat diet with a 45% caloric content originating from fat for 16 weeks showed increased body weight (t = 7.076; df = 21; p<0.001, Fig 1A), a higher percentage of adipose mass (11.59; df = 21; p<0.001; Fig 1B) and a relative proportional decrease in lean mass (t = 11.34; df = 21; p<0.001; Fig 1C) compared to mice fed on a low-fat diet.

### 3.2 Diet-induced obese mice exhibit visceral hypersensitivity

**Fig 2:**
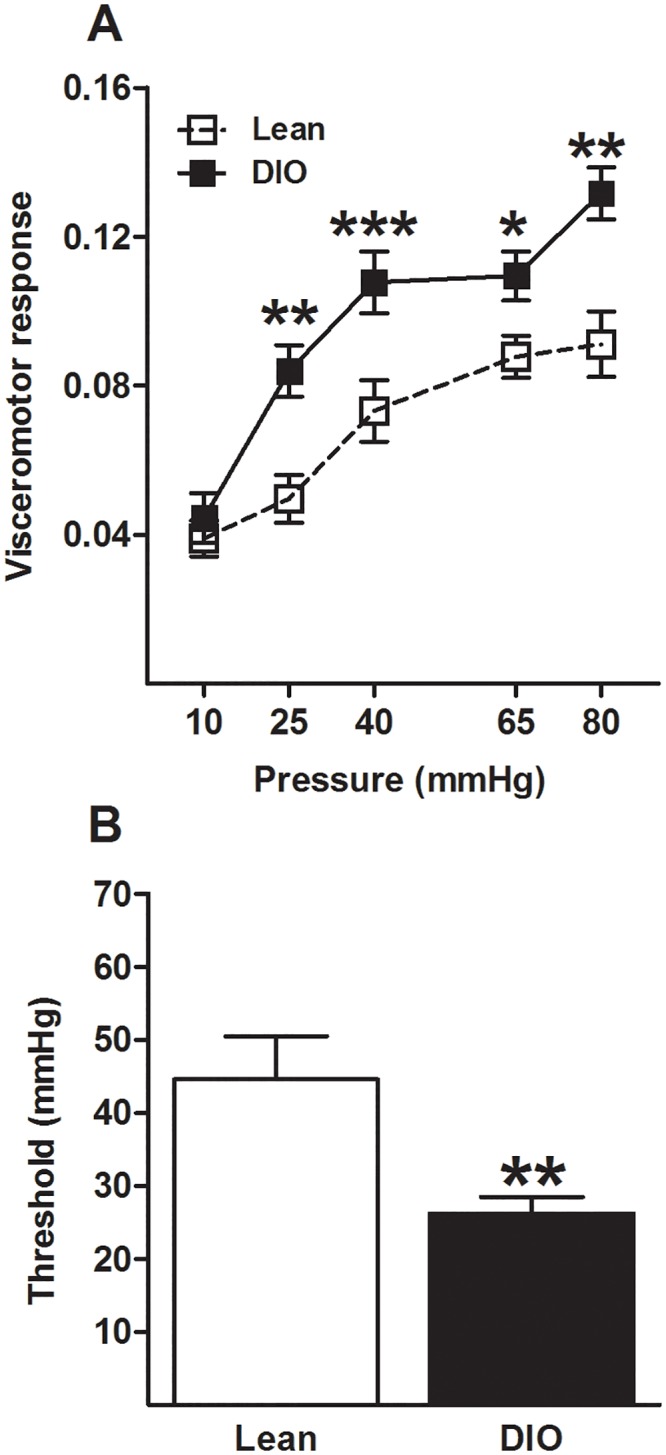
Diet-induced obese mice present with visceral hypersensitivity. In DIO mice visceral pain responses are increased (A) and similarly, the pain threshold is decreased (B) compared to lean animals. * p<0.05; ** p<0.01; *** p<0.001; independent sample t-test or repeated measures 1-way analysis of variance followed by Bonferroni’s post-hoc test; baseline response to CRD was not different between individual groups/conditions; data represent mean ± standard error of the mean (SEM).

Emerging evidence suggests that mice fed on a high-fat diet (HFD) exhibit characteristic signs of painful diabetic neuropathy [32, 33] including tactile allodynia, thermal hypoalgesia and nerve conduction deficits. Hence, we evaluated using CRD whether exposure to HFD modulates visceral pain. Mice fed on a HFD (DIO mice) over 16 weeks showed increased pain responses to CRD (diet: F(1,15) = 49.379; p<0.001, Fig 2A) along with a decreased pain threshold (t = 2.913, df = 22, p<0.01, Fig 2B) when compared with mice fed on a LFD (lean mice). A separated cohort of mice fed on a standard rodent diet also underwent CRD. No differences were observed on visceral sensitivity when compared with mice fed on a LFD (data not shown).

### 3.3 Exposure to high-fat diet is associated with increased protein expression of TLR4 in the prefrontal cortex, hippocampus and the lumbar region of the spinal cord

**Fig 3:**
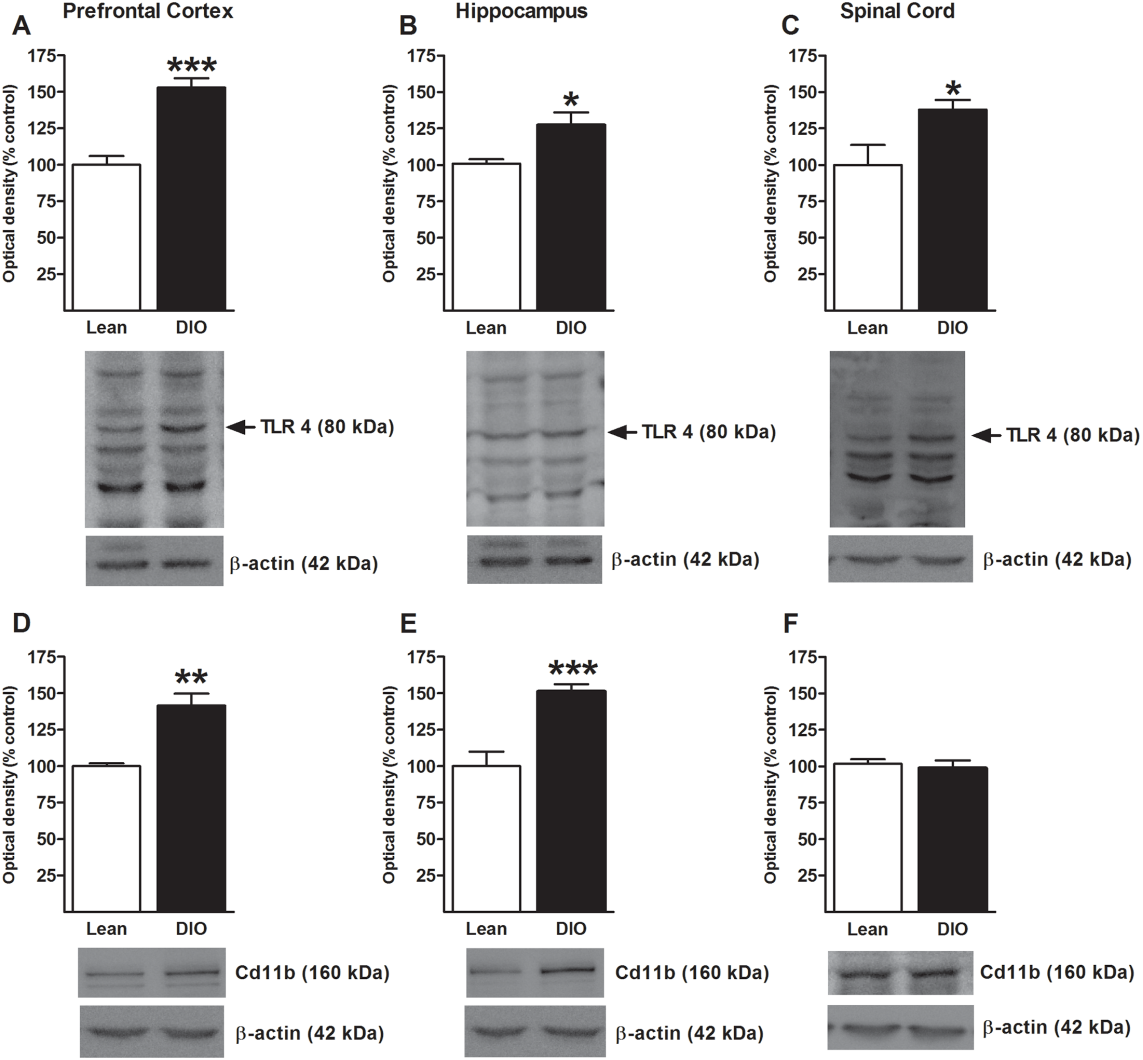
Increased TLR4 expression and microglia activation in the prefrontal cortex, hippocampus and the lumbar region of the spinal cord in DIO mice. Western blot analysis for TLR4 and Cd11b (microglial marker) in the prefrontal cortex, hippocampus and the lumbar region of the spinal cord. TLR4 is significantly increased in the prefrontal cortex in DIO mice (A), hippocampus (B) and spinal cord (C). Moreover, microglia expression (Cd11b) is significantly increased in DIO in the prefrontal cortex (D) and hippocampus (E), but not in the lumbar region of the spinal cord (F) when compared with lean mice. * p<0.05; ** p<0.01; *** p<0.001; independent sample t-test; data represent mean ± standard error of the mean (SEM) expressed as percentage of the control group.

Mice exposed to HFD show peripheral and central alterations in TLR4 expression levels [6, 10, 12] linked to the development of obesity. In turn, TLR4-deficient animals are resistant to the development of DIO [17, 18]. We have recently demonstrated that the modulation of visceral pain via TLR4 is associated with expression changes within the prefrontal cortex and also the hippocampus [19]. The PFC and hippocampus are two areas involve in the sensory, emotional and cognitive aspects of pain processing [21, 34]. In order to investigate whether HFD is involved in the modulation of TLR4 expression in these brain areas, TLR4 expression was measured using western blot. Protein levels of TLR4 were increased in DIO mice in the prefrontal cortex (t = 6.02; p<0.001, Fig 3A) and the hippocampus (t = 2.85; p<0.05, Fig 3B) when compared with lean mice. Other pathological pain states as inflammatory neuropathic and cancer pain have been associated with alterations of TLR4 at the spinal cord level [35–39]. However, spinal TLR4 expression has not been studied in a model of obesity-induced visceral hypersensitivity. In the lumbar region of the spinal cord, protein levels of TLR4 were significantly increased in DIO mice (t = 2.5; p<0.05, Fig 3C) when compared with lean mice. These data represent the first indication that TLR4 expression is altered at the spinal and supraspinal level, in two relevant brain structures associated with pain regulation, the prefrontal cortex and the hippocampus in DIO mice.

### 3.4 Exposure to high-fat diet is associated with microglia activation in the prefrontal cortex and hippocampus

TLR4 is mainly expressed in the CNS in microglia [15] and several studies have linked the etiology of pathological pain states [40, 41] including visceral hypersensitivity [42] with the activation of microglia in the CNS. To further investigate whether the increased expression of TLR4 observed in DIO mice is associated with microglia activation, protein levels of Cd11b, a microglial cell marker, were measured in the prefrontal cortex and the hippocampus. Protein levels of Cd11b were significantly increased in DIO mice in both regions, the prefrontal cortex (t = 4.3; p<0.01, Fig 3C) and the hippocampus (t = 4.97, p<0.001, Fig 3D) when compared to lean mice.

### 3.5 Exposure to high-fat diet increases peripheral TLR4 activity

**Fig 4:**
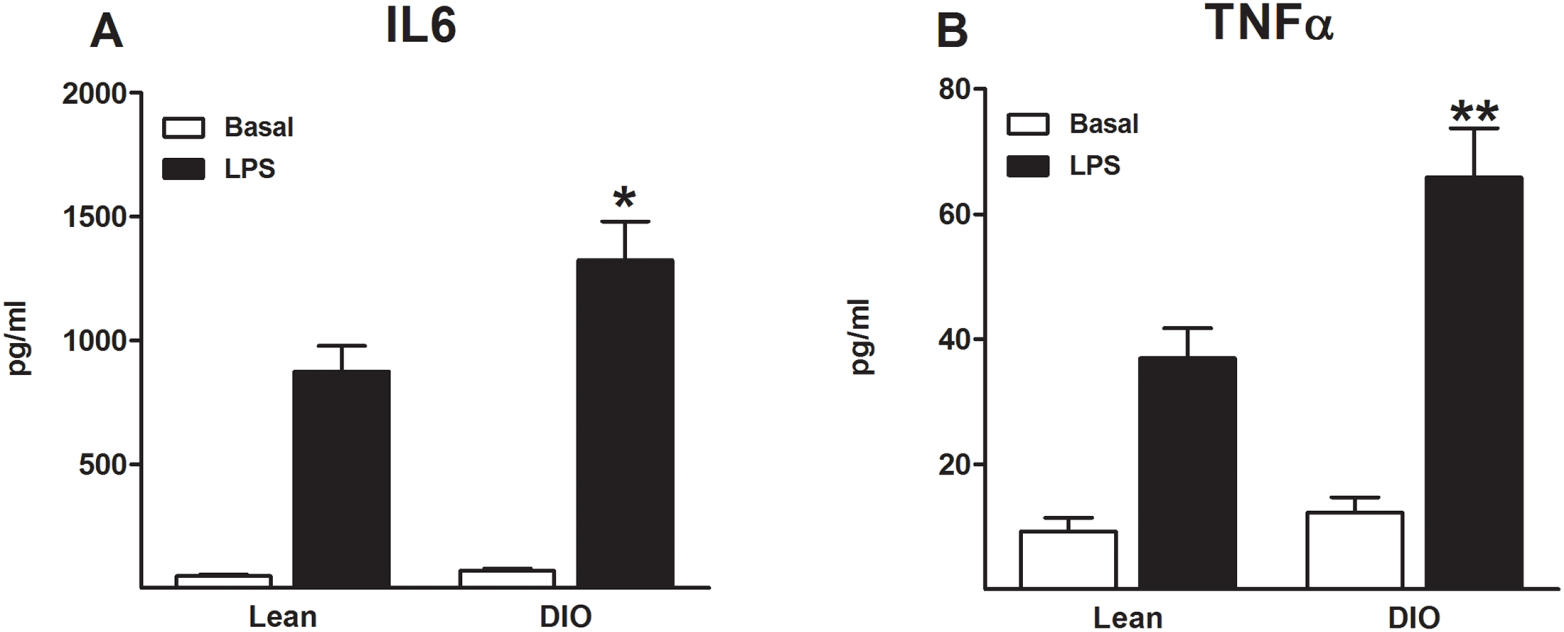
Increased cytokine levels following LPS spleen stimulation in DIO mice. Cytokine levels of IL6 and TNFα in spleen cell culture supernatant following stimulation with lipopolysaccharide (LPS; 1 μg/ml, 37°C, 24 h) were measured in mice fed on a low- or high-fat diet over 16 weeks. Exposure to HFD (DIO mice) increased significantly spleen levels of IL6 **(A)** and TNFα **(B)** after the stimulation with LPS, compared to mice fed on a LFD (lean mice). * p<0.05; ** p<0.01 vs. lean mice; 2-way analysis of variance followed by Bonferroni’s post-hoc test; data represent mean ± standard error of the mean (SEM).

In order to examine the impact of the HFD in TLR4 activity in the periphery, spleens from mice fed on the low- or high-fat diet were stimulated with 1μg/ml of lipopolysaccharide (LPS), a selective TLR4 agonist. IL6 and TNFα levels were measured in spleen culture supernatant. Mice exposed to a high-fat diet (DIO mice) exhibited an increased in the spleen levels of IL6 (LPS: F(1,34) = 123.8, p<0.001; diet: F(1,34) = 6.3, p<0.05; LPS x diet: F(1,34) = 5.3, p<0.05, Fig 4A) and TNFα (LPS: F(1,34) = 65.1, p<0.001; diet: F(1,34) = 10.0, p<0.01; LPS x diet: F(1,34) = 6.7, p<0.05, Fig 4B) compared to mice exposed to a low-fat diet (lean mice). No differences were observed in the levels of IL6 and TNFα in the unstimulated spleen preparations (basal) between mice exposed to a low or a high-fat diet.

### 3.6 TAK-242, a TLR4 antagonist, counteracts the visceral hypersensitivity phenotype in DIO mice

**Fig 5:**
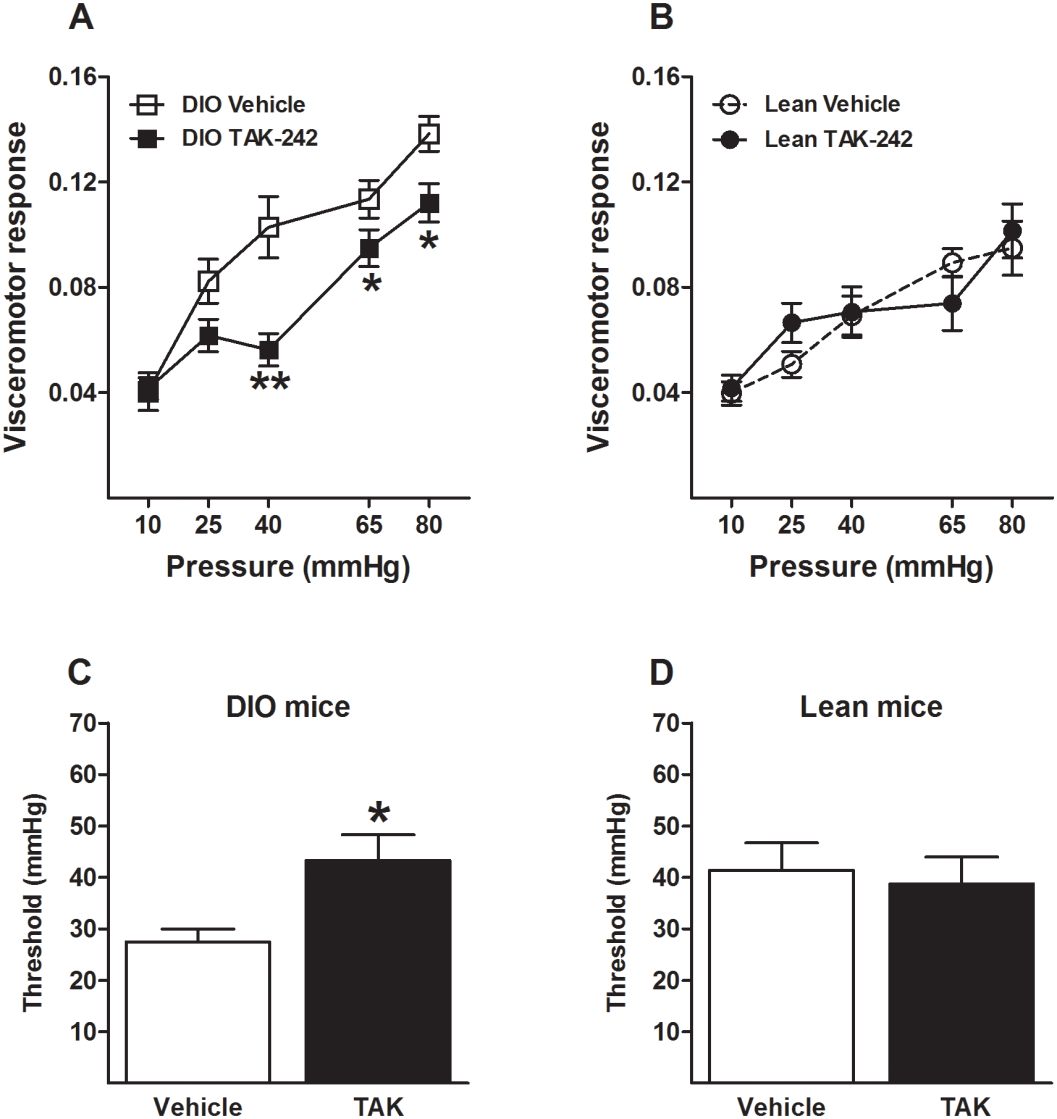
TAK-242 reverses the visceral hypersensitivity phenotype in DIO mice. Administration of the selective TLR4 antagonist TAK-242 (i.v. 10mg/kg) significantly reduces visceral pain responses in DIO (A) but not in lean mice (B). Moreover, the antagonist normalizes the decreased pain threshold in DIO mice (C) without affecting the pain threshold in lean mice (D). * p<0.05; ** p<0.01; independent sample t-test or repeated measures 2-way analysis of variance followed by Bonferroni’s post-hoc test; baseline response to CRD was not different between individual groups/conditions; data represent mean ± standard error of the mean (SEM).

TAK-242, a selective TLR4 antagonist, is a very small molecule and a highly lipophilic [30]. However, it is not known if TAK-242 could cross the brain blood barrier. In a previous study, we have shown that TAK-242 modulates visceral pain sensation in mice with functional TLR4 when administrated peripherally and centrally [19]. To further confirm that the visceral hyperalgesic phenotype present in DIO mice is directly associated with TLR4 alterations, TAK-242 was administered peripherally to mice exposed to the low or high-fat diet over 16 weeks. A single dose of this compound prominently decreased pain responses in visceral hypersensitive DIO mice (diet: F(1,31) = 25.48, p<0.001; treatment: F(1,31) = 17.41, p<0.001; diet x treatment: F(1,31) = 15.73, p<0.001, Fig 5A), whereas TAK-242 had no effect in animals on a low-fat diet (Fig 5B). Along with the decreased visceral pain responses, TAK-242 administration significantly increased the pain threshold in DIO mice (t = 2.155; p<0.05, Fig 5C) without affecting the pain threshold in lean mice (Fig 5D).

## Discussion

The present study examined the modulatory function of TLR4 in visceral pain in a model of diet-induced obesity. Mice fed on a high-fat diet show an obese phenotype characterized by increased body weight and percentage of adipose mass consistent with previous results from our group [23]. Our results demonstrate that long-term exposure to high-fat diet induces visceral hypersensitivity. This finding is in agreement with a recently published study showing that mice exposed to high-fat diet over 12 weeks exhibited visceral hyperalgesia [43]. It is important to note that there is a growing literature on the impact of the high-fat diet on nociception in rodents. These studies suggest that the consumption of fat-rich diets could impact the onset and progression of diabetic neuropathy symptoms including sensory and motor nerve deficits [32, 33, 44]. In addition, visceral hypersensitivity has been reported in a model of diabetic neuropathy in rats [45] and in diabetic patients [46].

In parallel with changes in visceral perception, diet-induced obese mice exhibit increased TLR4 expression within the CNS, in the prefrontal cortex (PFC), the hippocampus and in the lumbar region of the spinal cord. Our results are consistent with studies showing central alterations in TLR4 expression linked to the development of obesity [12]. The fact that visceral hypersensitivity is associated with increased expression of TLR4 in the PFC and hippocampus, two relevant-brain areas involved in visceral pain modulation [47–49], is in agreement with our previous report showing the modulatory function of TLR4 within the PFC in a model of stress-induced visceral hypersensitivity [19]. In addition, the superficial layers of the spinal dorsal horn have also been associated with the modulation of abdominal pain [50, 51]. In the present study, DIO mice also showed increased levels of expression of TLR4 in the spinal cord. Thus, TLR4 could exert a regulatory function in processing painful stimuli in this model of diet-induced visceral hypersensitivity. Our data are consistent with others studies showing central [35–39, 52] and peripheral [53, 54] alterations of TLR4 associated with several pathological pain states such as inflammatory, neuropathic and cancer pain. As TLR4 is primarily expressed within the CNS in microglial cells [15], further DIO mice showed enhanced CD11b expression in the PFC and the hippocampus, thus our results are in line with several studies supporting microglia activation as an important factor in the etiology of visceral pain [42, 55, 56]. By contrast, no changes in microglia levels were found in the lumbar region of the spinal cord in DIO mice. As TLR4 is also expressed on astrocytes [19], further studies are needed to evaluate whether the expression of glial cells in the PFC and hippocampus are regulated in mice exposed to fat-rich diets compared to lean mice. Along with these central changes, our results indicate increased TLR4 activity in the periphery in DIO mice, leading to an enhanced LPS-induced release of IL6 and TNFα from isolated spleenocytes. These findings are in agreement with other studies showing peripheral alterations of TLR4 following long-term exposure to high-fat diet [10, 12]. In our study, no differences were observed in the levels of IL6 and TNFα in the unstimulated spleens between mice exposed to a low or a high-fat diet. However, similar results have been observed in others studies when the profile of expression of cytokines in different tissues are measured in mice exposed to high-fat diet [11]. Pathological pain is viewed as an inflammatory state, both, in the periphery and in the CNS [57]. Furthermore, the initiation and maintenance of chronic pain states in animal models are associated with TLR4 activation and the subsequent release of several pro-inflammatory cytokines [35, 37, 39, 52, 53, 58]. Thus, pro-inflammatory cytokines such as IL-6 and TNFα, released following TLR4 activation with LPS could contribute to the visceral hyperalgesic phenotype in DIO mice. It is important to note that long-term exposure to a high-fat diet increased levels of circulating LPS [6, 10]. Therefore, enhanced plasma levels of LPS could lead to a constant release of pro-inflammatory cytokines through TLR4 activation. Other possible ligands of TLR4 linked to the consumption of high-fat diets include saturated fatty acids [12–14] and heat shock protein 70mRNA [59]. These molecules can bind to TLR4 [60] triggering the release of pro-inflammatory cytokines and as a result, visceral hypersensitivity.

Moreover, it is important to consider that several experimental reports have linked the exposure to high-fat diet with inflammation in the gastrointestinal tract associated to changes in gut microbiota [10, 61–66]. Further, clinical evidence reveals that dietary factors affect gut microbiota. Alterations in the balance between beneficial and harmful bacteria that reside in the gut are associated with various disease states. In fact, diet-induced gut microbiota disruption is a contributing factor in the development of functional gastrointestinal disorders like irritable bowel syndrome (IBS), as well as systemic diseases like obesity and diabetes [67]. In addition, it is well documented from both, clinical [68, 69] and preclinical studies [70, 71] the direct link between increased visceral pain perception and gut microbiota alterations in IBS, in which visceral hypersensitivity is as a core symptom.

To further demonstrate that the alterations in TLR4 observed in our study are directly mediating the visceral hyperalgesic phenotype present in DIO mice, we administered TAK-242, a selective TLR4 antagonist in mice following the exposure to a low- or high-fat diet. Our results confirm that the blockage of TLR4 completely counteracts the diet-induced visceral hypersensitivity, without affecting visceral sensitivity in lean mice. These results are consistent with our previous report showing the effect of TAK-242 blunting the visceral hyperalgesic phenotype present in chronic stress mice [19] and with a recently published report showing the analgesic properties of this compound in a model of neuropathic pain in rats [72]. The lack of effect observed in lean mice is not due to a floor effect associated with the CRD method used in the study. Additionally, several studies have shown the effect of blocking TLR4 in animal models of pathological pain. These reports conclude that the hyperalgesic phenotype in animal models of neuropathic pain is prevented [73] and also reversed [74] following the blockage of TLR4.

Taken together with our previous study, these data indicate that exposure to high-fat diet induce visceral hypersensitivity along with an increased TLR4 expression at central and peripheral level. Moreover, the selective pharmacological blockade of TLR4 with TAK-242 counteracts the hypersensitive phenotype in an animal model of diet-induced obese. Thus, blocking TLR4 may be a potential strategy to treat visceral hypersensitivity. Given the clinical availability of TAK-242, human trials are warranted to test the efficacy of TLR4 antagonists in functional gastrointestinal disorders and systemic diseases associated with a visceral hyperalgesic phenotype, such as IBS and diabetes.
